# Complete Genome Sequence of the Soil Bacterium *Pseudomonas kribbensis* Strain 46-2^T^

**DOI:** 10.1128/MRA.01161-18

**Published:** 2018-11-29

**Authors:** Haeyoung Jeong, Eugene Rha, Haseong Kim, Seung-Goo Lee

**Affiliations:** a Infectious Disease Research Center, Korea Research Institute of Bioscience and Biotechnology (KRIBB), Daejeon, Republic of Korea; b Synthetic Biology and Bioengineering Research Center, Korea Research Institute of Bioscience and Biotechnology (KRIBB), Daejeon, Republic of Korea; c Department of Biosystems and Bioengineering, KRIBB School of Biotechnology, University of Science and Technology (UST), Daejeon, Republic of Korea; Louisiana State University

## Abstract

Pseudomonas kribbensis is a novel species belonging to the Pseudomonas fluorescens intrageneric group of the genus Pseudomonas. Herein, we report the complete genome sequence of strain 46-2^T^, isolated from garden soil in Daejeon, South Korea. The 6.32-Mb chromosome contains 5,626 coding sequences with a G+C content of 60.55%.

## Abstract (precis)

Pseudomonas kribbensis is a novel species belonging to the Pseudomonas fluorescens intrageneric group of the genus Pseudomonas. Herein, we report the complete genome sequence of strain 46-2^T^, isolated from garden soil in Daejeon, South Korea.

## ANNOUNCEMENT

Bacterial strains belonging to the genus Pseudomonas, one of the most diverse genera, are ubiquitous and versatile microorganisms with a variety of metabolic capabilities. This genus is divided into two intrageneric groups (IGs), IG Pseudomonas aeruginosa and IG Pseudomonas fluorescens, based on multilocus sequence analysis employing four housekeeping genes (16S rRNA, *gyrB*, *rpoB*, and *rpoD*) (1). Pseudomonas kribbensis (2) was reported to be a novel species that is closely related to the Pseudomonas koreensis subgroup (P. fluorescens IG). We sequenced and analyzed the genome of the strain 46-2^T^, which was originally isolated from garden soil in Daejeon, Republic of Korea, as previously reported (2). For genomic DNA isolation, a single colony of P. kribbensis 46-2^T^ was inoculated in a 50-ml bioreactor tube with 5 ml of Luria broth and incubated at 30°C for 16 h in a shaking incubator. The cultured broth was transferred to a 50-ml conical tube, and the cells were harvested by centrifugation at 13,000 to 16,000 × *g* for 2 min. Then, we followed the instructions of the Wizard genomic DNA purification kit (Promega, Madison, WI, USA) which was used for the DNA isolation. Whole-genome sequencing of P. kribbensis 46-2^T^ was performed on a PacBio RS II platform (Pacific Biosciences, Menlo Park, CA, USA) using P6-C4 chemistry at the National Instrumentation Center for Environmental Management (Seoul, Republic of Korea).

For the creation of sequencing libraries, we used the PacBio DNA template prep kit version 3.0 according to the manufacturer’s instructions. Thereafter, 76,632 reads totaling 1.03 Gb (186.8× redundancy and *N*_50_ read length of 17,768 bp) were assembled into one contig of 6,335,277 bp in length using the Canu assembler version 1.5 (3) with the parameter genomeSize = 6.3m, which was subsequently circularized using the Circlator “all” command in version 1.5.1 (4). Residual errors were corrected by running two consecutive rounds of the RS_Resequencing.1 protocol using SMRT Analysis version 2.3. The final sequence consists of a chromosome of 6,324,282 bp with a G+C content of 60.55%.

Genome annotation was performed using the NCBIs Prokaryotic Genome Annotation Pipeline (PGAP) version 4.5 (5). The genome sequence encodes 5,626 coding sequences. It also encodes 6 rRNA gene clusters and 74 tRNAs. IslandViewer (6) predicted 85 genomic islands that do not include any virulence- or resistance-related genes, which sums to 862,993 bp, collectively. Nine secondary metabolite biosynthetic clusters were also identified using antiSMASH version 4.0 (7), including a gene cluster for bananamides (8) and mangotoxin-like compounds (9).

To identify the most closely related genome based on overall genome similarity, whole-genome average nucleotide identity (gANI) values were calculated using dRep version 2.0.5 (10) with the parameters –S_algorithm gANI –sa 0.95 across all publicly available Pseudomonas sp. genomes downloaded from the NCBI RefSeq database. A cluster consisting of P. fluorescens Pf0-1 (RefSeq assembly accession number GCF_000012445) and MS82 (RefSeq assembly accession number GCF_003055645) and Pseudomonas sp. DR 5-09 (RefSeq assembly accession number GCF_001655595) was closely related to 46-2^T^ (gANI, ∼91%), whereas P. koreensis LGM 21318^T^ was distantly related to 46-2^T^ (gANI, 87.4%). This observation is discordant with that in a previous report that categorized 46-2^T^ in the P. koreensis species group based on neighbor-joining phylogenetic analysis of four concatenated housekeeping genes (2). This disparity highlights that the use of genome sequence information and correct species labeling of the submitted genomes are crucial for comparative analyses. We speculate that the strain with the complete genome sequence can contribute to the research on the Pseudomonas sp. microbial community.

### Data availability.

The complete genome sequence of P. kribbensis 46-2^T^ has been deposited at DDBJ/ENA/GenBank under the accession number CP029608. Raw sequencing data files were deposited in the NCBI SRA under the accession number SRR7976405.
